# Potential risk of SARS-CoV-2 infection among people handling linens used by COVID-19 patients before and after washing

**DOI:** 10.1038/s41598-022-18945-8

**Published:** 2022-09-02

**Authors:** Retsu Fujita, Hitomi Kurosu, Masataro Norizuki, Takayuki Ohishi, Aya Zamoto-Niikura, Masaaki Iwaki, Keiko Mochida, Hirotaka Takagi, Toshihiko Harada, Kenji Tsushima, Tetsuya Matsumoto, Ken-Ichi Hanaki, Motoyuki Sugai, Takuya Yamagishi

**Affiliations:** 1 Graduate School of Medicine, International University of Health and Welfare, 4-1-26, Akasaka, Minato-ku, Tokyo, 107-8402 Japan; 2 Antimicrobial Resistance Research Center, National Institute of Infectious Diseases, 1-23-1 Toyama, Shinjuku, Tokyo 162-8640 Japan; 3 Bureau of International Health Cooperation, National Center for Global Health and Medicine, 1-21-1 Toyama, Shinjuku, Tokyo 162-8655 Japan; 4 Department of Infection Control and Prevention, Saiseikai Yokohama City Eastern Hospital, 3-6-1 Shimosueyoshi, Tsurumiku, Yokohama, Kanagawa 230-8765 Japan; 5 Management Department of Biosafety , Laboratory Animal, and Pathogen Bank, National Institute of Infectious Diseases, 1-23-1 Toyama, Shinjuku, Tokyo 162-8640 Japan; 6 International University of Health and Welfare Narita Hospital, 852 Hatakeda, Narita, Chiba 286-8520 Japan

**Keywords:** Microbiology, Environmental sciences, Infectious diseases

## Abstract

The risk of SARS-CoV-2 infection when people handle linens is uncertain. We examined the presence of SARS-CoV-2 on linens, in the air, and on personal protective equipment (PPE) to assess potential infection risk among individuals who handle linens used by SARS-CoV-2-infected people. Patients in a hospital and an accommodation facility who tested positive for SARS-CoV-2 participated in this study in 2020. Linen samples before washing or disinfection, rinse water after washing or disinfection, air in the workplace at the hospital and an accommodation facility, and the PPE worn by linen-handling people were tested for SARS-CoV-2 RNA and viable viruses. Among 700 samples from 13 SARS-CoV-2-infected participants and their surrounding environment, SARS-CoV-2 RNA was detected from 14% (52/362) of the linens used by COVID-19 patients (cycle threshold [Ct] value: 33–40). SARS-CoV-2 RNA was detected from 8% (2/26) of rinse water after washing or disinfection, from 15% (16/104) of air samples in the workspace, and from 10% (5/52) of gowns worn by linen-handling people, all with high Ct values (> 36). No SARS-CoV-2 was isolated from any samples. The potential risk of SARS-CoV-2 infection from handling linens used by SARS-CoV-2-infected people exists but appears to below.

## Introduction

The coronavirus disease (COVID-19) pandemic has threatened public health in unprecedented ways in the twenty-first century. According to the World Health Organization, by January 2022, 289 million people had been infected and more than 5.4 million had died of COVID-19^1^. Severe acute respiratory syndrome coronavirus 2 (SARS-CoV-2), which causes COVID-19, is transmitted via droplets, aerosols, and contact^2^. The mean duration of viral RNA shedding from the upper respiratory tract of COVID-19 patients is reported to be 17 days, and transmissibility can last for approximately 9 days after the onset of infection^3^. SARS-CoV-2 adhering to environmental surfaces is infectious for approximately 3 days, and there is a risk of contact transmission from contaminated environmental surfaces^4–7^. A study of a COVID-19 outbreak on a cruise ship detected SARS-CoV-2 RNA from bedding and other linens used by infected people^8^. Similarly, several subsequent studies observed that viral RNA can be isolated from pillowcases, bedsheets, and other bedding used by patients^9–11^. Currently, there are no standard practices for how to safely handle and clean linens used by SARS-CoV-2-infected people. Several authorities recommended that the warmest water possible should be used to wash a COVID-19 patient’s clothes^12–15^, and there is a report that suggests that surfactants used in detergents and softeners may deactivate the virus^16^. However, evidence about the risk of SARS-CoV-2 infection when handling linens used by COVID-19 patients is limited. Thus, we examined the presence of SARS-CoV-2 on linens, in rinse water after washing or disinfection, in air, and on personal protective equipment (PPE) following linen changes to assess potential risk of viral spread among individuals who handle and wash linens used by SARS-CoV-2-infected participants.

## Bullet methods

### Study design and participants

This study was designed as an open-label, randomized controlled trial. Participants were registered from September 16, 2020 to November 19, 2020 at a hospital admitting symptomatic COVID-19 patients and an accommodation facility housing asymptomatic SARS-CoV-2-infected people. Symptomatic COVID-19 patients or patients with comorbidities were admitted to the hospital after confirming a positive polymerase chain reaction (PCR) test result from their nasopharyngeal specimen. Asymptomatic SARS-CoV-2-infected people were sent to the accommodation facility after a positive antigen test result. The antigen test was performed at the quarantine station at a nearby airport. At the hospital, participants stayed in negative-pressure rooms (5.5 air changes per hour; area, 14.5 m^2^; and volume, 36 m^3^). At the accommodation facility, the participants stayed in private rooms (air changes per hour, unknown; area, 12 m^2^; and volume, 30 m^3^).

The presence of SARS-CoV-2 RNA on linens before and after washing or disinfection was evaluated on the day that participants were admitted to the hospital or facility (day 1) and on day 3 after admission. Also, to assess the potential infection risk among people handling linens in the rooms where COVID-19 patients were staying, air and PPE were sampled and analyzed for the presence of SARS-CoV-2 RNA.

### Randomization

The participants were assigned one of the five types of washing and disinfection methods shown below for each facility and laundry opportunity (days 1 and 3) using static simple randomization. Blinding was not performed.

### Assessment of linen contamination and evaluation of washing or disinfection

The linens used by SARS-CoV-2-infected participants (sheet; pillowcase; duvet; cloths (upper and lower part); cloths bottom; bath towel; and face towel) were changed and sampled after 1, 3, 5, and 7 days of use to test for SARS-CoV-2 RNA. Linens were made of either cotton or a cotton-polyester blend. The linens collected on days 1 and 3 were washed or disinfected using one of the five randomly assigned methods: (1) washing in tap water (15–25 °C), (2) washing in tap water (15–25 °C) with a commercially available polyoxyethylene laundry detergent (Attack Zero^®^, Kao Corporation, Tokyo, Japan), (3) washing in tap water (15–25 °C) with a commercially available softener (Humming^®^, Kao Corporation), (4) disinfection by immersion for 30 min in a 250-ppm sodium hypochlorite solution, and (5) disinfection by immersion for 10 min in hot water (80 °C). The presence of SARS-CoV-2 RNA was tested from rinse water samples.

Samples from linens used by the SARS-CoV-2-infected participants were collected using 10 × 87-mm sterile flocked plastic swabs (Eiken Chemical, Japan). Before sample collection, the swabs were moistened with viral transport medium containing 2% heat-inactivated fetal bovine serum (FBS), 100 μg/mL gentamicin, 50 μg/mL amphotericin B, 8 μg/mL tylosin, and 10 μg/mL levofloxacin (VTM; Hanks’ balanced salt solution). The moistened swabs were inserted into 21.9 × 93.6-mm tubes containing 8 mL VTM. Samples were collected by wiping the swab in three directions over a 100 × 100 mm^2^ area near where the participant’s face had been on the sheet, pillowcase, and duvet: on the collar for the nightgown top, near the feet for the nightgown bottom, and near the middle for the bath and face towels. After washing or disinfecting the linens, 500 mL of rinse water was collected in sterilized bottles and stored at 4 °C. Prior to analysis, the samples were filtered with a drip filter to remove debris, and then the filtered samples were weighed and 5% polyacrylic acid (MW 25 kDa) dissolved in 0.4 M NaOH to a final concentration of 0.13% was added. After repeating mixing and standing at room temperature for 30 min, the samples were centrifuged at 11,000×*g* and the pellets were collected. Finally, the supernatants were obtained by resuspending the pellets in 2 mL of PBS (-) and centrifuging at 6000 rpm for 3 min.

### Presence of airborne SARS-CoV-2

To assess the risk of droplet and airborne transmission while people handled SARS-CoV-2-contaminated linens, air samples were collected before and during or after linen changes. A Sartorius MD8 Airscan sample device (Sartorius AG, Göttingen, Germany) was placed at a height of 100 cm and 50 cm away from the bed. Sterile gelatin filters (80-mm diameter; 3-μm pore size, T3 phage-capture rate 99.94%; Sartorius AG) were used to filter 2000 L of air at a flow rate of 50 L/minute. The patients remained in the room during linen changes and while air was sampled. After air sampling, the gelatin membrane filter was fragmented and inserted into a 100-mL centrifugation tube with 5 mL VTM. The filter was dissolved by shaking at 37 °C for 15 min and then stored at − 80 °C until RNA extraction.

To assess the potential risk of contact transmission while people handled SARS-CoV-2-contaminated linens, samples were collected from the surfaces of their PPE immediately after changing the linens. Fifty-two samples were collected from each PPE item (N95 mask, goggles, upper gown, and lower gown) by wiping the swab in three directions over a 50 × 50-mm^2^ area: on front surface of the mask, front surface of the goggles, near the neck for the gown top, and near the feet for the gown bottom.

### SARS-CoV-2 RNA detection and virus isolation

All samples were transported to the National Institute of Infectious Diseases (NIID) and examined using the real-time reverse transcriptase (rtRT) PCR method developed by Shirato et al.^16^ Viral RNA was extracted from samples, including nasopharyngeal, linen, and PPE swabs, using a MagMAX CORE nucleic acid purification kit (Thermo Fisher Scientific, Tokyo, Japan) on the KingFisher Flex purification system (Thermo Fisher Scientific) according to the manufacturer’s instructions: each 60-µL RNA elution was prepared from 200-µL samples. The rtRT-PCR mixture, including the N2 primer set, was prepared using a QuantiTect Probe PCR kit (QIAGEN, Venlo, Nederland) in a final volume of 20 µL. This comprised 5 µL extracted RNA, 0.5 µM forward primer, 0.7 µM reverse primer, and 0.2 µM TaqMan probe. The reaction was performed, using a LightCycler 480 system (Roche, Basel, Switzerland) with reverse transcription at 50 °C for 30 min followed by denaturation at 95 °C for 15 min. Next, 45 cycles of amplification were performed using a thermal cycling profile of 95 °C for 15 s and 60 °C for 1 min. The viral copy numbers in patient specimens were calculated using a standard curve established by the NIID. The limit of detection of cycle threshold (Ct) values was set at 40 based on a report by Buchan et al. and the NIID protocol^17^.

The specimens used for virus isolation were prepared as inoculation material that had undergone sterile filtration using a centrifuge filter unit (Ultrafree-MC, 0.22 μm; Merck Millipore, Burlington, MA, USA). Vero E6/TMPRSS2 cells (5% FBS DMEM) cultured in 12-well plates were inoculated with 100 μL of the specimen and cultured at 37 °C and 5% CO_2_ for 7 days to check for a cytopathic effect (CPE). A total of 100 µL of the first culture supernatants was transferred to a new monolayer cell plate and cultured under the same conditions as described above. After three serial passages, we judged positivity/negativity by CPE.

### Statistical analysis

For demographic data, summary statistics were shown as medians (interquartile range or range), frequency distributions, or proportions. Meteorological parameters were shown as mean (± standard deviation). The difference in RNA detection proportions between the accommodation facility and hospital was evaluated by Fisher’s exact test. Longitudinal changes in RNA detection proportions were assessed with the Cochran-Armitage test, and a *p* value less than 0.05 was considered statistically significant. A 95% confidence interval was used to determine whether there were statistically significant differences in the percentages of positive samples among the different groups of cleaning and disinfection methods. All statistical analyses were performed with SAS V.9.4 (SAS Institute, Cary, NC, USA).

### Ethical approval

We explained the risks and benefits of the study to all participants and obtained their informed consent. All methods were performed in accordance with the relevant guidelines and regulations, and this study was approved by the Institutional Review Boards of the NIID (approval No. 1167) and International University of Health and Welfare (approval No. 20-Nr-066).

## Results

**Figure 1:**
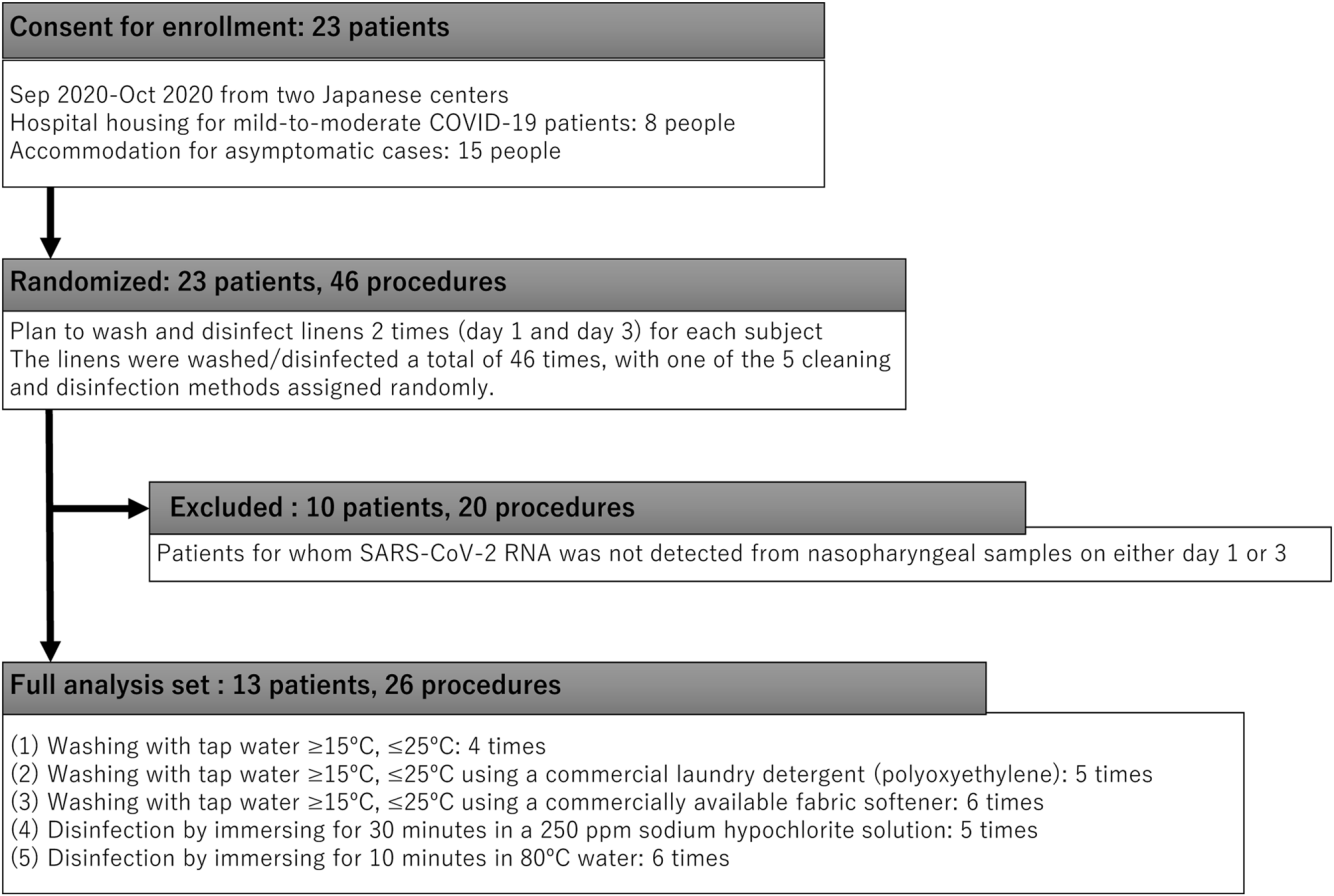
Enrollment, assignment to cleaning or disinfection methods and exclusion of the participants in the study.

Among the 23 patients enrolled, 13 (seven from the hospital and six from the accommodation facility) tested positive for SARS-CoV-2 RNA on day 1 or day 3 and were included in the analysis (Fig. 1). Among these 13 participants, seven were women (54%) and the median age was 46 (interquartile range 33–55) years (Table 1). Seven participants including one at the accommodation facility (54%) were symptomatic during the study period. Their median interval from symptom onset to hospitalization or entering the accommodation facility was 1 day (range, 0–3 days). All symptomatic participants had mild symptoms, and none had moderate or severe pneumonia based on the National Institutes of Health classification^18^. The rooms were continuously air-conditioned in both the hospital and the accommodation facility. Temperature and humidity were measured once a day at 12:00 noon, the mean (± standard deviation) values of which were 24.2 ± 1.2 °C and 47.8 ± 14.2%, respectively. During the study period, the health conditions of the researchers (healthcare workers) who were involved in the study, which included sample collection, were mutually evaluated, but no symptoms were revealed.

**Table 1:** Participant demographic characteristics and clinical symptoms.

	Hospital (n = 7)	Accommodation (n = 6)	Total (n = 13)
Age, median (interquartile range)	55 (43–61)	29.5 (23–46)	46 (33–55)
Sex, female (%)	3 (43)	4 (67)	7 (54)
**Underlying conditions (%)**			
Respiratory disease	1 (14)	0	1 (8)
Liver disease	1 (14)	0	1 (8)
Diabetes mellitus	1 (14)	0	1 (8)
Allergic disease	0	1 (17)	1 (8)
**Clinical symptoms on linen sampling (%)**			
Number of patients with symptoms	6 (86)	1 (17)	7 (54)
**Type of symptoms**			
Cough	5 (71)	0	5 (39)
Fever	1 (14)	1 (17)	2 (15)
Smelling or tasting disorder	2 (29)	0	2 (15)
Fatigue	1 (14)	0	1 (8)
**Results of nasopharyngeal rRT-PCR on linen sampling**			
Ct value on Day 1, median (range)	24.6 (18.9–30.9)	30.1 (18.8–37.8)	25.8 (18.8–37.8)
Ct value on Day 3, median (range)	30.7 (21.6–31.2)	30.5 (26.9–40.0)	30.7 (21.6–40.0)

In total, 700 samples were tested for the presence of SARS-CoV-2 RNA: 362 from linens, 26 from rinse water, 104 from air, and 208 from PPE. SARS-CoV-2 RNA was detected in 52 of the 362 linen items tested (14%, Table 2). At both facilities, SARS-CoV-2 RNA was detected in all linen types. The detection frequency ranged from 12 to 21% for bedding (sheet, pillowcase, duvet, and cloths (upper, lower)) and between 6 and 8% for bath and face towels. No significant differences were observed in the detection proportions with respect to the type of linen (Fisher’s exact, sheets *p* = 0.31, pillow case *p* = 0.72, duvet cover *p* = 0.67, clothes upper *p* = 0.73, clothes lower *p* = 1.00, bath towel *p* = 1.00, face towel *p* = 1.00), or overall linen (*p* = 0.65) between facility residents and hospitalized patients. The RNA detection proportions decreased significantly over time in the hospital (Cochran-Armitage, *p* < 0.01), although not in the accommodation facility (*p* = 0.25) or overall (*p* = 0.19).

**Table 2:** SARS-CoV-2 RNA test results of the linens used by SARS-CoV-2-positive patients.

	Day 1		Day 3		Day 5		Day 7		Total		
	SARS-CoV-2 RNA positive*	Samples tested	SARS-CoV-2 RNA positive	Samples tested	SARS-CoV-2 RNA positive	Samples tested	SARS-CoV-2 RNA positive	Samples tested	SARS-CoV-2 RNA positive		Samples tested
**Hospital**											
Sheets	1 (14%, 0–58)	7	2 (29%, 4–71)	7	1 (14%, 0–58)	7	0 (0%, 0–41)	7	4 (14%, 4–33)		28
Pillow case	3 (43%, 10–82)	7	1 (14%, 0–58)	7	0 (0%, 0–41)	7	0 (0%, 0–41)	7	4 (14%, 4–33)		28
Duvet cover	1 (14%, 0–58)	7	1 (14%, 0–58)	7	1 (14%, 0–58)	7	1 (14%, 0–58)	7	4 (14%, 4–33)		28
Clothes, upper	3 (43%, 10–82)	7	1 (14%, 0–58)	7	1 (14%, 0–58)	7	1 (17%, 0–64)	6	6 (22%, 8–41)		27
Clothes, lower	2 (29%, 4–71)	7	1 (14%, 0–58)	7	1 (14%, 0–58)	7	1 (17%, 0–64)	6	5 (19%, 6–37)		27
Bath towel	1 (14%, 0–58)	7	1 (14%, 0–58)	7	0 (0%, 0–41)	7	0 (0%, 0–41)	7	2 (7%, 1–24)		28
Face towel	1 (14%, 0–58)	7	1 (14%, 0–58)	7	0 (0%, 0–41)	7	0 (0%, 0–41)	7	2 (7%, 1–24)		28
Sub total	12 (24%, 16–45)	49	8 (16%, 7–30)	49	4 (8%, 3–23)	49	3 (6%, 2–19)	47	26 (13%, 9–19)		194
**Accommodation**											
Sheets	1 (17%, 0–64)	6	1 (17%, 0–64)	6	3 (50%, 12–88)	6	2 (33%, 4–78)	6	7 (29%, 13–53)		24
Pillow case	1 (17%, 0–64)	6	0 (0%, 0–46)	6	2 (33%, 4–78)	6	2 (33%, 4–78)	6	5 (21%, 7–44)		24
Duvet cover	1 (17%, 0–64)	6	1 (17%, 0–64)	6	1 (17%, 0–64)	6	0 (0%, 0–46)	6	2 (8%, 1–28)		24
Clothes, upper	1 (17%, 0–64)	6	0 (0%, 0–46)	6	1 (17%, 0–64)	6	2 (33%, 4–78)	6	4 (17%, 5–39)		24
Clothes, lower	1 (17%, 0–64)	6	1 (17%, 0–64)	6	2 (33%, 4–78)	6	1 (17%, 0–64)	6	5 (21%, 7–44)		24
Bath towel	0 (0%, 0–46)	6	1 (17%, 0–64)	6	0 (0%, 0–46)	6	0 (0%, 0–46)	6	1 (4%, 0–22)		24
Face towel	0 (0%, 0–46)	6	0 (0%, 0–46)	6	1 (17%, 0–64)	6	0 (0%, 0–46)	6	2 (8%, 1–28)		24
Sub total	5 (12%, 4–26)	42	4 (10%, 3–23)	42	10 (24%, 12–39)	42	7 (17%, 7–31)	42	26 (15%, 10–22)		168
**Total**											
Sheets	2 (15%, 2–45)	13	3 (23%, 5–54)	13	4 (31%, 9–61)	13	2 (15%, 2–45)	13	11 (21%, 11–35)		52
Pillow case	4 (31%, 9–61)	13	1 (8%, 0–36)	13	2 (15%, 2–45)	13	2 (15%, 2–45)	13	9 (17%, 8–30)		52
Duvet cover	2 (15%, 2–45)	13	2 (15%, 2–45)	13	2 (15%, 2–45)	13	1 (8%, 0–36)	13	6 (12%, 4–23)		52
Clothes, upper	4 (31%, 9–61)	13	1 (8%, 0–36)	13	2 (15%, 2–45)	13	3 (25%, 5–57)	12	10 (20%, 10–33)		51
Clothes, lower	3 (23%, 5–54)	13	2 (15%, 2–45)	13	3 (23%, 5–54)	13	2 (17%, 2–48)	12	10 (20%, 10–33)		51
Bath towel	1 (8%, 0–36)	13	2 (15%, 2–45)	13	0 (0%, 0–25)	13	0 (0%, 0–25)	13	3 (6%, 1–16)		52
Face towel	1 (8%, 0–36)	13	1 (8%, 0–36)	13	1 (8%, 0–36)	13	0 (0%, 0–25)	13	4 (8%, 2–19)		52
Total	17 (19%, 11–28)	91	12 (15%, 9–24)	91	14 (15%, 9–24)	91	10 (11%, 6–20)	89	52 (14%, 11–18)		362

Fisher’s exact test showed no significant difference in the RNA detection proportion for all linens between the accommodation and hospital.

The Cochran–Armitage test showed a significant difference in the trend of RNA detection proportion in the hospital (p = 0.0018) but not in the accommodation (p = 0.25) and total result (p = 0.19).

*The number of positive samples (percentage of positive samples, 95% confidence intervals for the percentages).

SARS-CoV-2 RNA was not detected in rinse water after washing with tap water, disinfecting with sodium hypochlorite, or disinfecting with 80 °C water (Table 3). However, SARS-CoV-2 was detected in one of five samples (20%; Ct value, 40) after washing with laundry detergent (polyoxyethylene) and in one of six samples (17%; Ct value, 37) after washing with fabric softener. No virions were isolated in any of the samples.

**Table 3:** SARS-CoV-2 RNA test results of rinse water after washing/disinfection.

Cleaning methods	All linens		Linens with SARS-CoV-2 RNA*	
	SARS-CoV-2 RNA positive**	Samples tested	SARS-CoV-2 RNA positive	Samples tested
Water	0 (0%, 0–60)	4	0 (0%, 0–60)	0
Detergent	1^†^ (20%, 1–72)	5	1^†^ (100%, 3–100)	1
Softener	1^↑^ (17%, 0–64)	6	1^↑^ (25%, 1–82)	4
80 °C, 10 min	0 (0%, 0–52)	5	0 (0%, 0–70)	3
NaClO 250 ppm	0 (0%, 0–46)	6	0 (0%, 0–98)	1

*Linens in which SARS-CoV-2 RNA was detected before washing or disinfection.

**The number of positive samples (percentage of positive samples, 95% confidence intervals for the percentages).

^†^Cycle threshold value = 40. The viral isolation result was negative.

^↑^Cycle threshold value = 37. The viral isolation result was negative.

Table 4 shows the results of the tests on the air samples collected before and during or after the changing of linens. SARS-CoV-2 RNA was detected in 16 samples, which were more frequently detected during or after the changing of linens (10 samples, 18%) than before the changing of linens (six samples, 11%); however, the difference was not statistically significant (*p* = 0.42). SARS-CoV-2 RNA was detected more frequently in the accommodation facility (17% before and 29% after the changing of linens) than in the hospital (7% before and 11% after the changing of linens); however, the difference also was not statistically significant (*p* = 0.06). In five cases, the air sample before changing of the linen was negative but turned positive during or afterward. The Ct values were all ≥ 36, and no virions were isolated after three serial passages. SARS-CoV-2 RNA was detected in six of the 208 PPE samples, five from the lower gown (10%) and one from an upper gown (2%; Table 5). No viral RNA was detected on N95 masks or goggles. The Ct values of all six samples that tested positive for SARS-CoV-2 RNA were ≥ 37, and no virions were isolated after three serial passages.

**Table 4:** SARS-CoV-2 test results of air samples collected before and during/after changing linens.

	SARS-CoV-2 RNA positive*^†^		Change of Ct value	Sample sets tested
	Before linen collection (baseline)	During/after linen collection	Detected/increased	
Hospital	2 (7%, 1–24)	3 (11%, 2–28)	1	28
Accommodation	4 (17%, 5–37)	7 (29%, 13–51)	5	24
Total	6 (11%, 4–22)	10 (18%, 9–30)	6	52

*All Ct values detected were ≥ 36. The viral isolation result was negative.

^†^The number of positive samples (percentage of positive samples, 95% confidence intervals for the percentages).

Fisher’s exact test showed no significant difference in the RNA detection rate of air samples before and during/after linen exchange (p = 0.42) and between the accommodation and hospital (p = 0.06).

**Table 5:** SARS-CoV-2 test results of personal protective equipment after changing the linens.

Items	SARS-CoV-2 RNA positive*^†^	Samples tested
**Hospital**		
N95 respirator	0 (0%, 0–12)	28
Gown, upper	1 (4%, 0–18)	28
Gown, lower	3 (11%, 2–28)	28
Goggles	0 (0%, 0–12)	28
**Accommodation**		
N95 respirator	0 (0%, 0–14)	24
Gown, upper	0 (0%, 0–14)	24
Gown, lower	2 (8%, 1–27)	24
Goggles	0 (0%, 0–14)	24
**Total**		
N95 respirator	0 (0%, 0–7)	52
Gown, upper	1 (2%, 0–10)	52
Gown, lower	5 (10%, 3–21)	52
Goggles	0 (0%, 0–7)	52

*All Ct values detected were ≥ 37, and the viral isolation result was negative.

^†^The number of positive samples (percentage of positive samples, 95% confidence intervals for the percentages).

## Discussion

This study monitored the presence of SARS-CoV-2 RNA on linens of SARS-CoV-2-infected people before and after washing, in the air during linen collection, and on PPE of workers changing linens. The goal of the study was to better understand the potential infection risk among people who change linens used by SARS-CoV-2-infected people and to determine if the virus remains on linens after washing and/or disinfection. SARS-CoV-2 RNA was detected on linens used by SARS-CoV-2-infected people and gowns of workers who changed the linens. SARS-CoV-2 RNA detection increased from baseline during or after changing the linens but all were with a high Ct value (> 36), which indicates that although droplet or airborne SARS-CoV-2 transmission while changing the linens is possible, the risk is low. The finding that SARS-CoV-2 RNA was not detected from N95 masks or goggles worn by people who changed the linens, and negative results on virus isolation tests, support this hypothesis. Minimal SARS-CoV-2 RNA was detected in the rinse water after washing linens with laundry detergent or fabric softener. The samples had high Ct values and negative results on virus isolation tests indicating that the risk of SARS-CoV-2 infection from handling washed linens would be low.

Several studies have examined the relationship between Ct values and transmissibility. According to previous reports, it is difficult to culture viruses from specimens with Ct values > 35^19–21^. Moreover, studies discussing the clinical interpretation of high Ct values have expressed negative views when assessing the transmissibility of viral RNA detected with Ct values in the high 30 s^22^. In our study, SARS-CoV-2 RNA was detected on the linens used by SARS-CoV-2-infected people, on PPE worn by the people who had changed the linens, in air samples where the linens were changed, and in the rinse water after the washing of linens. The Ct values of the contaminated linens ranged from 33 to 40. Thus, although the risk of contact transmission from contaminated linens could exist, it is unlikely that the SARS-CoV-2 RNA detected on PPE, in the air, and on washed linens in our study would contribute to SARS-CoV-2 transmission.

The results of our study confirmed the presence of SARS-CoV-2 on linens used by SARS-CoV-2-infected people. However, there was no difference in the detection frequency between the hospital and the accommodation facility suggesting that a similar level of linen contamination occurs regardless of disease severity. Previous studies have reported that viral shedding does not differ significantly between asymptomatic and symptomatic people^23,24^ and that the risks of transmission from asymptomatic/pre-symptomatic and symptomatic patients are similar^25,26^.

The frequency of linen contamination over time confirmed a downward trend only in the hospital but not in the accommodation facility. A possible reason for the lack of a clear trend in the accommodation facility is that viral shedding in COVID-19 patients is highest from 2 days before symptom onset to the day after onset^25,27,28^. Thus, as the accommodation facility housed mostly asymptomatic people, there may have been a wide variation in the duration since infection. In our study, viral RNA with Ct values up to the low 30 s, which suggests infectiousness, continued on the linens used by SARS-CoV-2-infected people up to 5 days after study enrollment. The mean period of viral RNA shedding in COVID-19 patients is 17 days after onset in the upper respiratory tract^3^, and SARS-CoV-2 adhering to environmental surfaces remains infectious for approximately 3 days^4,5^. Although we did not completely evaluate changes in the RNA detection proportions over time, or the attenuation of infectiousness, our results suggest that caution is needed when handling linens used by COVID-19 patients at least within 5 days of symptom onset or sample collection.

The results of the air sampling confirmed that viral particles float in the air before and after changing the linens. As it was reported that SARS-CoV-2 RNA exists in the air in hospitals and in urban environments around COVID-19 patients^20,29–31^, we assumed that SARS-CoV-2 RNA would be detected before changing the linens. Although no statistically significant differences were observed, the frequency of SARS-CoV-2 RNA detection was higher during or after changing the linens than before. This suggests the possibility that handling linens caused viral particles to disperse into the air. Nonetheless, the Ct values of all detected viral RNA were ≥ 36, and the virus isolation test results were negative. Also, no viral RNA was detected from N95 and goggles. Therefore, the risk of droplet or airborne transmission from inhaling SARS-CoV-2 while changing contaminated linens might be low.

On comparing washing and disinfection methods, SARS-CoV-2 RNA was detected in two samples each of rinse water after washing with laundry detergent (polyoxyethylene) and a fabric softener (grade 4 ammonium). It was suggested that viruses are inactivated by surfactants in detergents or softeners^16^. On the basis of the finding that all Ct values were high (> 37) and no viral RNA was detected in rinse water after washing, we conclude that the risk of SARS-CoV-2 transmission from washed linens is low. Moreover, if people practice proper hand hygiene and use PPE and it is ensured that linens are retrieved and transported safely, it seems unnecessary to recommend the disinfection procedures currently adopted by many countries^12–15^.

This study had several limitations. First, we only examined asymptomatic patients and symptomatic patients with mild upper respiratory tract symptoms and those with severe disease were not included. The period of viral shedding does not differ greatly between people with mild disease and those with moderate or severe diseases^23,28^; however, medical procedures that can spread the virus such as suctioning are performed more often on patients with moderate or severe pneumonia. Studies of the degree of linen contamination during the care of patients with moderate or severe disease are warranted. Second, Ct values for asymptomatic participants were slightly higher than those for symptomatic participants, and although the exact timing of exposure was unclear, it may take more time for asymptomatic participants between exposure and identification (testing) than for symptomatic participants. However, we confirmed that the linens were contaminated by virus RNA and the influence of the different viral shedding levels from each participant were limited. Third, although this study was designed as a randomized controlled trial to compare washing and disinfection methods, it was not a clinical trial with a verification design in which power and sample size were set in advance. Fourth, we only tested one detergent and one softener. Commercially available laundry detergents and softeners vary widely in ingredients and forms, making it difficult to choose representative cleaning agents and methods. Fifth, because participants remained in the room when linens were changed, air sampling could have detected viral particles from patients’ exhalations. However, the detection proportion increased after the handling of linens, which indicates that linen changing scattered virus particles. Sixth, only the surface of the workers’ N95 masks was swabbed, and viral RNA inside the masks was not measured. Finally, the findings of our study were based on the wild variant and may not be applicable to other viral variants, although variation in environmental viral stability between variants appears to be low^32^.

In conclusion, this study found SARS-CoV-2 RNA on the linens used by SARS-CoV-2-infected people could be without viable viruses or viral RNA with low Ct value after washing by water, detergent, or softener. The possibility of airborne transmission and contact transmission of SARS-CoV-2 during or after the handling of linens could not be fully ruled out, but transmission by both routes appears to be less likely. The kinds of PPE healthcare workers need to wear depends on the acceptability of the risk of SARS-CoV-2 transmission, and our findings help to provide information useful to assess this risk (Supplementary Information).

## Supplementary Information

Supplementary Information.

## Data Availability

The data collected in this study, including de-identified participant data and the data dictionary, are available to researchers through corresponding author Dr. Takuya Yamagishi upon reasonable request. These data will be available for a period of 6 months to 3 years after publication. Data requests require a methodologically sound proposal, a data access agreement and approval by the local ethics committee.
