# Supplementary material for: Potential risk of SARS-CoV-2 infection among people handling linens used by COVID-19 patients before and after washing
Source: Sci Rep. 2022 Sep 2;12:14994. doi: 10.1038/s41598-022-18945-8 (PMC9438874; doi:10.1038/s41598-022-18945-8)
Supplement: Supplementary file 1 — Supplementary Information. [file 41598_2022_18945_MOESM1_ESM.docx]

**SUPPLEMENTARY MATERIAL: METHODS**

**Linen cleaning and disinfection methods**

1. **Washing with tap water ≥15°C, ≤25°C**

- The linens were washed in a commercially available household washing machine, and just before completing the final step, 500 mL of rinse water was collected in a sterilized bottle.
- Asymptomatic case accommodation facility: MCW-C50 (AQUA)

The washing machine was operated in standard mode (washing step 8 minutes, first rinsing step 1 minute, and second rinsing step 3 minutes). The washing machine held 50 L of water, and approximately 110 L of water was used for all the steps.

- Hospital: ES-GE7D (SHARP)

The washing machine was operated in standard mode (washing step: 8 minutes, first rinsing step: 1 minute, and second rinsing step: 3 minutes). The washing machine held 55 L of water, and approximately 120 L of water was used for all the steps.

**2. Washing in tap water ≥15°C and ≤25°C using commercially available laundry detergent**

- - The linens were washed with the same machines and the same steps as described in (1) above using 20 mL of commercially available laundry detergent (amount recommended by the retailer).
  - Main ingredients of the laundry detergent (Attack zero, Kao Corporation, Japan)
    - Surfactants: polyoxyalkylene alkyl ether, hydroxy alkane sulfonate, alkene sulfonate, fatty acid salt, cumene sulfonate, polyoxyalkylene alkyl ether sulphate ester salt, and polyoxyethylene alkyl ether
    - Stabilizers: phenoxyethanol, butyl-carbitol
    - Dispersants: acrylate-based copolymers, cellulose polymers
    - Preservative

**3. Washing in tap water ≥15°C and ≤25°C using commercially available fabric softener**

- - The linens were washed in the same machines and with the same steps as in scenario (1) above using 30 mL of commercially available fabric softener (amount recommended by the retailer).
  - Main ingredients of the fabric softener (Humming, Kao Corporation, Japan)
    - Surfactants: ester-type dialkyl ammonium salt, polyoxyethylene alkyl ether, and sorbitan fatty acid ester
    - Stabilizer: ethylene glycol
    - Fiber lubricant: dimethicone
    - Scent
    - Viscosity adjuster: calcium chloride
    - pH adjuster: citric acid
    - Foam adjuster: silicone
    - Preservative

1. **Disinfection by immersion in 250 ppm sodium hypochlorite solution for 30 minutes**

- 85 mL 6% sodium hypochlorite solution was added to 20 L of tap water at ≥15°C and ≤25°C to create a 250-ppm sodium hypochlorite solution.
- The linens were immersed and disinfected for 30 minutes after confirming that the residual chlorine concentration was in the range of 250–270 ppm with a chlorine concentration-measuring instrument.
- After disinfection using a chlorine concentration-measuring instrument, it was confirmed that the residual chlorine concentrations remained higher than 200 ppm.
- The linens were rinsed in approximately 100 L of tap water, and a sterilized bottle was used to collect 500 mL of the rinse water.

1. **Disinfection by immersion in 80°C water for 10 minutes**

- The linens were disinfected by immersing them in a 40-L insulated container filled with tap water that was heated to 80–85°C.
- After disinfection, a thermometer was used to confirm that the water temperature had stayed above 80°C.
- The linens were rinsed in approximately 100 L of tap water, and a sterilized bottle was used to collect 500 mL of the rinse water.
